# Integrating genomic insights and artificial intelligence to elucidate lipoprotein(a)-mediated risk in cardio-renal syndrome

**DOI:** 10.1097/MS9.0000000000004323

**Published:** 2025-11-13

**Authors:** Aanchal Rohra, Sakshi Rohra, Mahnoor Fatima, Muhammad Talha, Ubaid Ur Rehman

**Affiliations:** a Department of Medicine, Liaquat University of Medical and Health Sciences, Jamshoro, Pakistan; b Department of Medicine, Isra University, Hyderabad, Pakistan; c Department of Medicine, Rajshahi Medical University, Ladies hostel, Shaheed Ziaur Rahman Medical College, Bogura, Bangladesh; d Department of Medicine, King Edward Medical University, Lahore, Pakistan

*To the Editor,*

Cardio-renal syndrome (CRS) refers to the bidirectional relationship between the heart and kidneys, where failure of one precipitates a decline in the other. The two organs have common risk factors such as dyslipidemia, hypertension, and diabetes but express dysfunction differently. Patients usually present with fluid overload, decreased urine output, and dyspnea. Patient history, laboratory tests, and imaging are helpful in diagnosis. Treatment typically involves correcting the underlying cause, maintaining fluid balance, and managing concurrent conditions. In critical situations, renal replacement therapy is indicated^[1]^. This is in line with the TITAN Guidelines on the need for transparency in artificial intelligence (AI) use in health care^[2]^.

Genetic differences in the Lipoprotein (a) (LPA) gene primarily determine lipoprotein (a) [Lp(a)] levels and are an independent risk factor for cardiovascular conditions such as coronary heart disease and myocardial infarction. Increased Lp(a) also plays a role in CRS through mechanisms that compromise cardiac and renal function^[3]^. AI can be used to dissect big genomic data to determine genetic Lp(a) variants, including single-nucleotide polymorphisms and haplotypes that influence their levels. Machine learning algorithms make predictions of which variants pose thrombotic risk by evaluating genotype-phenotype associations. This would potentially allow for customized therapies and early interventions for patients presenting with unusual CRS presentations^[4]^.

A recent study determined a strong correlation between elevated serum Lp(a) and CRS since increased Lp(a) facilitates plaque accumulation that causes renal blood vessel narrowing, aggravating kidney failure and heart disease^[5]^. Investigators examined patients with coronary heart disease (CHD) or those at high risk, determining increased Lp(a) as greater than 50 mg/dL. A computer algorithm clustered patient information according to LDL cholesterol, status for CHD, history of family, and age and found that individuals with low LDL-C, no CHD, and no family history had the lowest Lp(a) levels, whereas CHD patients with high LDL-C and a pertinent family history had significantly higher levels. This highlights the utility of AI in identifying patients at risk of having high Lp(a)^[6]^.

Despite proven advantages of this approach, several limitations remain. To use AI techniques efficiently in healthcare, a coordinated process involving humans and computers is indispensable. It involves meticulous data acquisition, manual labeling, extraction, and normalization before beginning training using an appropriate dataset. The dataset also needs to be tested before it’s ready for use. Still, the results produced by AI are not conclusive. They require confirmation by specialists. Scarcity of data, leading to sparse practice, makes AI implementations challenging. It is difficult to keep the explainability and reliability of AI approaches transparent for health care professionals and patients^[7]^.

The confluence of cardiology, nephrology, and genomics provides fresh perspectives on intricate diseases such as CRS, especially with genetic influences such as Lp(a). There is a need to highlight how AI helps in better understanding the link between genes and their effects on disease and to support improved involvement of computer techniques in clinical practice. It calls for high-quality multicenter datasets and cross-sector synergies between health care and data science. Ultimately, the goal is to advance precision medicine in cardio-renal therapy^[4,7]^.

In conclusion, the use of AI in analyzing Lp(a) genetic variants to determine prothrombotic risk in patients with rare CRS is a breakthrough in disease prevention. Prospective trials and collaboration of geneticists, clinicians, and computer scientists are necessary to confirm the role of this technology, compare it with conventional methods, and make algorithms more practical. Regulatory and ethical frameworks must be set up to safeguard genetic information and make equitable access possible.

## Data Availability

Not applicable to this study.
